# Estimation of the Prevalence of Delayed Dispensing Among Opioid Prescriptions From US Surgeons and Dentists

**DOI:** 10.1001/jamanetworkopen.2022.14311

**Published:** 2022-05-27

**Authors:** Kao-Ping Chua, Jennifer F. Waljee, Michael A. Smith, Shreya Bahl, Romesh P. Nalliah, Chad M. Brummett

**Affiliations:** 1 Department of Pediatrics, Susan B. Meister Child Health Evaluation and Research Center, University of Michigan Medical School, Ann Arbor; 2 Department of Health Management and Policy, University of Michigan School of Public Health, Ann Arbor; 3 Section of Plastic Surgery, Department of Surgery, University of Michigan Medical School, Ann Arbor; 4 Michigan Opioid Prescribing Engagement Network, University of Michigan Medical School, Ann Arbor; 5 College of Pharmacy, University of Michigan, Ann Arbor; 6 University of Michigan School of Dentistry, Ann Arbor; 7 Division of Pain Medicine, Department of Anesthesiology, University of Michigan Medical School, Ann Arbor

## Abstract

**Importance:**

Dispensing of opioid prescriptions from dentists and surgeons more than 30 days after writing, or delayed dispensing, could be a potential indicator that opioids were used for reasons or during a time frame other than that intended by the prescriber. The prevalence of delayed dispensing is unknown. Whether laws can prevent delayed dispensing by shortening the maximum period between prescription writing and dispensing is also unknown.

**Objective:**

To estimate the prevalence of delayed dispensing among opioid prescriptions from surgeons and dentists, assess the maximum period US states allow between controlled substance prescription writing and dispensing, and evaluate whether laws shortening this period decrease delayed dispensing of opioid prescriptions from surgeons and dentists.

**Design, Setting, and Participants:**

In this cross-sectional analysis, data from the IQVIA Formulary Impact Analyzer (representing 63% of US prescriptions) were used to identify opioid prescriptions from surgeons and dentists dispensed from 2014 through 2019.

**Main Outcomes and Measures:**

Among opioid prescriptions dispensed in 2019, the proportion with delayed dispensing was calculated. Using legal databases, the maximum state-allowed period between controlled substance prescription writing and dispensing as of December 2019 was examined. Using a difference-in-differences design and 2014 to 2019 data, changes in delayed dispensing prevalence were evaluated among opioid prescriptions from surgeons and dentists after a Minnesota law was enacted in July 2019 precluding opioid prescription dispensing more than 30 days after writing. Control states allowed dispensing beyond this period.

**Results:**

In 2019, the database included 20 858 413 opioid prescriptions from surgeons and dentists for 14 789 984 patients; 8 582 029 (58.0%) were female. The mean (SD) patient age was 47.1 (19.3) years. Of prescriptions included, 194 452 (0.9%) had delayed dispensing. As of December 2019, the maximum period between drug writing and dispensing was 180 days in 18 and 43 states for Schedule II and III drugs, respectively. Compared with control states, Minnesota’s law decreased delayed dispensing prevalence by 0.22 percentage points (95% CI, −0.32 to −0.13 percentage points).

**Conclusions and Relevance:**

In this cross-sectional study, 194 452 opioid prescriptions from surgeons and dentists were dispensed more than 30 days after writing. To mitigate any prescription opioid misuse associated with delayed dispensing, policy makers could shorten the maximum period between writing and dispensing of opioid prescriptions from surgeons and dentists.

## Key Points

**Question:**

How often are opioid prescriptions from US surgeons and dentists dispensed more than 30 days after writing (delayed dispensing)?

**Findings:**

In this cross-sectional analysis of a national pharmacy database representing 63% of US prescriptions, 194 452 opioid prescriptions (0.9%) from surgeons and dentists in 2019 were dispensed more than 30 days after writing. The prevalence of delayed dispensing decreased after a Minnesota law was enacted to preclude dispensing of opioid prescriptions more than 30 days after writing.

**Meaning:**

These findings raise concerns that opioids prescribed by surgeons and dentists may sometimes be used for reasons or during a time frame other than that intended by the prescriber.

## Teaser

This cross-sectional study estimates the prevalence of opioid prescriptions from US surgeons and dentists dispensed more than 30 days after writing and evaluates changes as a result of US state laws enacted to shorten the maximum period between opioid prescription writing and dispensing.

## Introduction

According to the 2019 US National Survey on Drug Use and Health,^1^ 9.7 million Americans had past-year prescription opioid misuse, defined as the use of prescription opioids belonging to others or use of prescription opioids in a manner or for a reason other than prescribed. Although record current levels of US opioid overdose deaths are mostly driven by illicit opioids,^2^ prescription opioid misuse is a risk factor for illicit opioid use.^3,4^ Consequently, reducing prescription opioid misuse is an important step to prevent opioid overdose deaths from both prescription and illicit opioids.^5^

In surgery and dentistry, efforts to prevent prescription opioid misuse have focused on limiting unnecessary or excessive opioid prescribing after procedures,^6,7,8^ mandating the use of prescription drug monitoring programs to detect concerning opioid use patterns,^9^ and encouraging safe storage and disposal of unused opioids.^10^ However, few efforts have focused on preventing misuse of opioids from prescriptions dispensed well after the writing date. Current federal and state laws enable such delayed dispensing. Under the Controlled Substances Act (CSA), prescriptions for US Drug Enforcement Administration (DEA) Schedule III and IV controlled substances (eg, buprenorphine and tramadol) can be dispensed up to 6 months after they are written unless states have passed laws shortening this period.^11,12^ In contrast, the CSA does not regulate the maximum period between prescription writing and dispensing for Schedule II (eg, oxycodone) or V (eg, combinations of acetaminophen and low-dose codeine) controlled substances.^11,12^ Some states have passed laws shortening the maximum period between prescription writing and dispensing for Schedule II or V controlled substances.^13^ However, in states without such laws, this period is either unregulated or defaults to the maximum period allowed for prescriptions in general, assuming states regulate the latter period.

To our knowledge, the prevalence of delayed dispensing among opioid prescriptions from US surgeons and dentists has not been estimated. Moreover, recent data are lacking on the maximum period between writing and dispensing of controlled substance prescriptions allowed by each state.^13^ Finally, it is unknown whether laws shortening this period can decrease delayed dispensing of opioid prescriptions from surgeons and dentists.

To address these knowledge gaps, we conducted 3 analyses. First, in a cross-sectional analysis of a national pharmacy database, we identified opioid prescriptions from surgeons and dentists dispensed in 2019. We calculated the proportion dispensed more than 30 days after writing, a potential indicator that opioids were used for reasons or during a time frame other than that intended by the prescriber. Second, we searched legal databases to determine the maximum period between controlled substance prescription writing and dispensing allowed by each state as of December 31, 2019. Finally, to assess whether laws shortening this period can decrease delayed dispensing, we used a difference-in-differences design and data from the same national pharmacy database for the period 2014 to 2019 to evaluate changes in the prevalence of delayed dispensing among opioid prescriptions from surgeons and dentists after a July 2019 Minnesota law was enacted to preclude dispensing of opioid prescriptions more than 30 days after writing.^14^

## Methods

### Data Sources

Because the data used were deidentified, the University of Michigan Medical School Institutional Review Board exempted this cross-sectional study from review, and informed consent was waived. The study followed the Strengthening the Reporting of Observational Studies in Epidemiology (STROBE) reporting guideline.

The IQVIA Formulary Impact Analyzer is a national pharmacy database that includes 63% of US prescriptions. Key data elements include prescription writing and dispensing dates, prescriber specialty, payment method (including cash), DEA schedule, and prescription type (refill vs original). A separate opioid prescription issued after a prior prescription ran out was considered original. The IQVIA database does not report diagnosis codes or patient race or ethnicity. Data sources for state laws were NABPLAW, a repository of pharmacy laws from the National Association of Boards of Pharmacy, and Westlaw Edge, a legal database from Thomson Reuters.^15,16^

### Sample

We identified all prescriptions for opioid analgesics dispensed in 2019 to a patient residing in 1 of 50 US states or the District of Columbia (referred to hereafter as a state). We used IQVIA’s market definition to identify opioid analgesics (eAppendix 1 in the Supplement). We limited prescriptions to those written by a surgeon or dentist, including general dentists, dental subspecialists, and oral and maxillofacial surgeons. We excluded prescriptions with missing or invalid data for the writing date. We further excluded refills, as the period between writing of the original prescription and dispensing of the refill would expectedly be long. For the difference-in-differences analysis of Minnesota’s law, we identified nonrefill opioid prescriptions from surgeons and dentists dispensed during 2014 to 2019. We limited analyses to prescriptions for Schedule II to IV opioids, the targets of the law. In all analyses, patients were allowed to have multiple prescriptions.

### Outcome

The outcome was delayed dispensing, defined as dispensing of prescriptions more than 30 days after writing. We chose this threshold for 2 reasons. First, it was consistent with Minnesota’s law. Second, opioid prescriptions from surgeons and dentists are usually prescribed for immediate management of acute postprocedural pain. As such, dispensing that occurs more than 30 days after writing might raise concerns that opioids were used for reasons or during a time frame other than that intended by the prescriber, which are both forms of prescription opioid misuse. Importantly, our goal was not to prove that delayed dispensing represented misuse; rather, we aimed to estimate the number of opioid prescriptions from surgeons and dentists with a potentially concerning dispensing pattern, information that could inform whether efforts to prevent any misuse associated with delayed dispensing may be warranted. For additional context, we used other cutoffs to describe the distribution of the period between opioid prescription writing and dispensing.

### Maximum Period Between Writing and Dispensing of Controlled Substance Prescriptions Allowed by Each State

We used the 2 legal databases to identify laws regulating the maximum period between writing and dispensing of controlled substance prescriptions in each state as of December 31, 2019 (methods and links to relevant state laws are provided in eAppendices 2 and 3 in the Supplement). If states lacked laws regulating this period for Schedule III or IV drugs, we set the maximum period to 6 months per the CSA. If states lacked laws regulating this period for Schedule II or V drugs, we assumed it was either restricted to the state’s maximum period allowed for prescriptions in general or unrestricted if the latter was also unregulated. We obtained information on laws regulating prescription dispensing in general through a separate search of the legal databases. Except for Minnesota’s law, which applied only to narcotics, laws applied to all drugs in the DEA schedule.

### Evaluation of Minnesota Law

We used the 2 legal databases to identify any state laws enacted during 2014 to 2019 that shortened the maximum period between writing and dispensing of controlled substance prescriptions. According to our research, Louisiana shortened this period to 90 days for Schedule II drugs in April 2015, New Mexico shortened it to 6 months for Schedule II drugs in October 2016, and Minnesota shortened it to 30 days for Schedule II to IV narcotics on July 1, 2019. Before July 2019, Minnesota did not regulate the maximum period between prescription writing and dispensing for any Schedule II drug but did restrict this period to 180 days for Schedule III to IV drugs. We chose to evaluate Minnesota’s law because few opioid prescriptions from surgeons and dentists are dispensed more than 90 days and 6 months after writing.

To evaluate whether Minnesota’s law decreased the prevalence of delayed dispensing among opioid prescriptions from surgeons and dentists, we conducted a difference-in-differences analysis.^17^ The preintervention period was January 1, 2014, to June 30, 2019. The postintervention period was July 1 to December 31, 2019. Of the other 50 states, we excluded 9 that restricted the maximum period between writing and dispensing of prescriptions for Schedule II, III, or IV drugs to 30 days or less. We also excluded Louisiana, New Mexico, and Florida, the last of which prohibited dispensing of prescriptions for Schedule II to III drugs more than 14 days after the date of a surgical procedure. The remaining 38 states were control states.

### Statistical Analysis

For opioid prescriptions dispensed in 2019, we used descriptive statistics to calculate the prevalence of delayed dispensing overall and among subgroups defined by patient characteristics, DEA schedule, extended-release/long-acting opioid status, surgical and dental subspecialty, and state. For additional context, we assessed the prevalence of delayed dispensing among opioid prescriptions written by other types of clinicians.

In the difference-in-differences analysis, we fitted a prescription-level linear regression model with state fixed effects, year-month fixed effects, and the interaction between an indicator for treatment group and postintervention period. The interaction term was the coefficient of interest. We used linear models to facilitate interpretation of coefficients as absolute percentage point changes in probability. The results were virtually identical when using logistic regression and calculating average marginal effects.^18^ To assess for parallel preintervention trends, we limited the sample to the preintervention period and fitted linear regression models with terms for month, an indicator for treatment group, and their interaction (ie, the slope difference). The models used robust SEs clustered at the state level.^19^ Analyses were performed with Stata 15.1 MP software (StataCorp) and 2-sided hypothesis tests (α = .05).

## Results

### Sample

The national pharmacy database contained 108 784 511 opioid prescriptions dispensed in 2019. Of these, 21 129 745 (19.4%) were from surgeons or dentists. The following prescriptions were excluded: 707 (0.003%) with missing or invalid data for prescription writing date and 270 625 refills (1.3%). The remaining 20 858 413 prescriptions were for 14 789 984 patients; 8 582 029 (58.0%) were female and 6 207 955 were male (42.0%). The mean (SD) patient age was 47.1 (19.3) years. Among the prescriptions in the sample, 12 666 995 (60.7%) were paid by commercial insurers, 15 933 181 (76.4%) were for Schedule II opioids, 13 214 637 (63.4%) were written by surgeons, and 7 644 046 (36.6%) were written by dentists.

### Prevalence of Delayed Dispensing

Among the 20 858 413 prescriptions included, the mean (SD) duration between writing and dispensing was 1.5 (7.5) days, with a median of 0 (25th-75th percentile, 0-0; range, 0-365) days. There were 16 286 319 prescriptions (78.1%) dispensed on the writing date vs 4 572 094 afterward (1-3 days, 2 925 840 [14.0%]; 4-14 days, 1 159 636 [5.6%]; 15-30 days, 292 166 [1.4%]; 31-90 days, 166 996 [0.8%]; 91-180 days, 25 563 [0.1%]; and 181-365 days, 1893 [0.01%]). Overall, 194 452 prescriptions (0.9%) were dispensed more than 30 days after writing (delayed dispensing), and 486 618 (2.3%) were dispensed more than 14 days after writing.

As shown in Table 1, the proportion of opioid prescriptions with delayed dispensing varied modestly by demographic characteristics. Among surgical subspecialties, this proportion ranged from 0.3% in pediatric surgery to 1.9% in ophthalmology. Among the 51 states, this proportion ranged from 0.1% to 1.9% (eAppendix 4 in the Supplement). The proportion of opioid prescriptions dispensed more than 30 days after writing was generally higher for other clinicians (5.6% for internal medicine specialists, 7.1% for family medicine clinicians, 6.3% for nurse practitioners, and 5.5% for physician assistants) than for surgeons and dentists (1.0% and 0.9%, respectively; eAppendix 5 in the Supplement).

**Table 1.** Prevalence of Delayed Dispensing Among Opioid Prescriptions From Surgeons and Dentists in 2019, Using IQVIA Formulary Impact Analyzer Data

Group	No. of prescriptions (% in sample)^a^	No. of prescriptions dispensed >30 d after writing (% in group)^b^
All prescriptions	20 858 413 (100)	194 452 (0.9)
Age group, y		
0-17	894 355 (4.3)	13 455 (1.5)
18-34	4 575 165 (21.9)	32 061 (0.7)
35-54	6 505 123 (31.2)	56 159 (0.9)
55-64	4 101 289 (19.7)	42 148 (1.0)
≥65	4 782 481 (22.9)	50 629 (1.1)
Sex		
Male	8 696 058 (41.7)	79 993 (0.9)
Female	12 156 709 (58.3)	114 413 (0.9)
Unknown	5646 (<0.1)	46 (0.8)
Census region		
Northeast	1 948 022 (9.3)	8914 (0.5)
Midwest	4 564 369 (21.9)	28 734 (0.6)
South	10 115 779 (48.5)	108 235 (1.1)
West	4 230 243 (20.3)	48 569 (1.1)
Payment method^c^		
Cash	1 401 394 (6.7)	11 164 (0.8)
Medicaid/other public insurance	2 633 169 (12.6)	15 105 (0.6)
Medicare	4 156 855 (19.9)	47 505 (1.1)
Commercial insurance	12 666 995 (60.7)	120 678 (1.0)
DEA schedule		
II	15 933 181 (76.4)	139 558 (0.9)
III	2 523 271 (12.1)	19 950 (0.8)
IV	2 357 389 (11.3)	34 466 (1.5)
V	44 572 (0.2)	478 (1.1)
Extended-release/long-acting opioid	124 786 (0.6)	4844 (2.5)
Prescriber specialty		
All surgeons	13 214 367 (63.4)	127 786 (1.0)
Cardiothoracic or thoracic	94 179 (0.5)	709 (0.8)
Colorectal	134 945 (0.6)	586 (0.4)
General	2 242 163 (10.7)	15 186 (0.7)
Hand	431 992 (2.1)	2173 (0.5)
Neurosurgery	509 714 (2.4)	9247 (1.8)
Obstetrics and gynecology	1 953 805 (9.4)	11 633 (0.6)
Ophthalmology	162 152 (0.8)	3019 (1.9)
Orthopedics	5 177 855 (24.8)	61 380 (1.2)
Otolaryngology	726 782 (3.5)	4536 (0.6)
Pediatric	63 774 (0.3)	171 (0.3)
Plastic surgery	684 505 (3.3)	8066 (1.2)
Transplant surgery	6281 (0)	35 (0.6)
Urology	890 581 (4.3)	9887 (1.1)
Vascular surgery	135 639 (0.7)	1158 (0.9)
All dentists	7 644 046 (36.6)	66 666 (0.9)
Oral maxillofacial surgery	5 578 807 (26.7)	36 686 (0.7)
Other dentists	2 065 239 (9.9)	29 980 (1.5)

Abbreviation: DEA, US Drug Enforcement Administration.

^a^ Column percentages of all prescriptions in the sample.

^b^ Row percentages of all prescriptions in the group.

^c^ Percentages do not total 100% because of rounding.

### Maximum Period Allowed Between Writing and Dispensing of Controlled Substance Prescriptions

**Figure 1.**
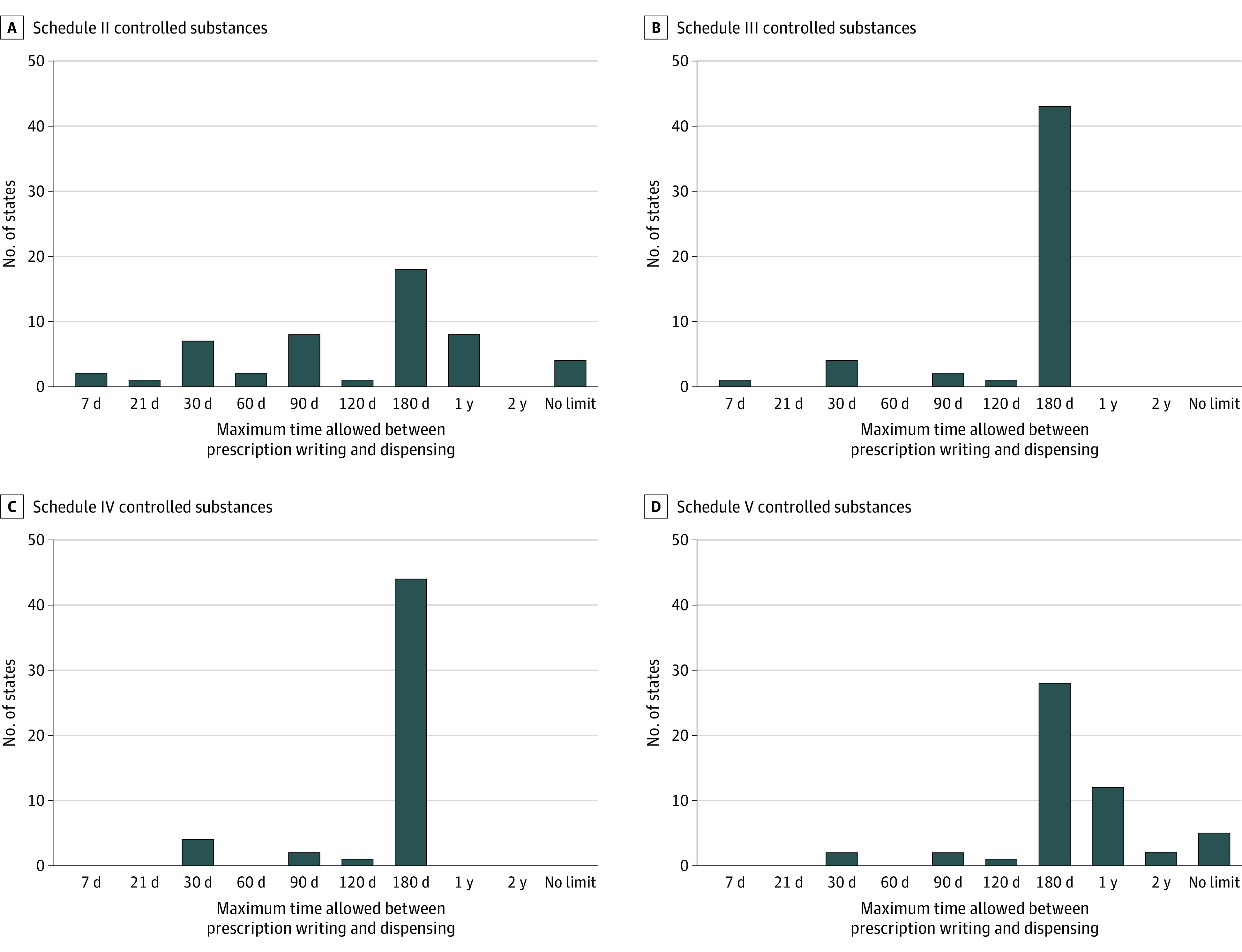
Maximum Period Between Writing and Dispensing of Controlled Substance Prescriptions Allowed by Each State as of December 31, 2019 Minnesota had a law that restricted the maximum period to 30 days only for Schedule II to IV opioids. We counted Minnesota in the number of states with a maximum period of 30 days for Schedule II to IV drugs.

As shown in Table 2 and Figure 1, 9 states limited the maximum period between writing and dispensing of Schedule II prescriptions to 30 days or less, 11 limited it to 60 to 120 days, 18 limited it to 180 days, and 8 limited it to 1 year. Four states did not limit this period, whereas Minnesota limited it to 30 days for opioids only. For Schedule III drugs, 4 states limited the maximum period between writing and dispensing of prescriptions to 30 days or less, 3 limited it to 90 to 120 days, and 43 limited it to 180 days; Minnesota limited this period to 30 days for opioids only. For Schedule IV drugs, 3 states limited the maximum period between writing and dispensing of prescriptions to 30 days, 3 limited it to 90 to 120 days, and 44 limited it to 180 days; Minnesota limited this period to 30 days for opioids only. For Schedule V drugs, 2 states limited the maximum period between writing and dispensing of prescriptions to 30 days, 3 limited it to 90 to 120 days, 28 limited it to 180 days, 12 limited it to 1 year, and 1 limited it to 2 years. Five states did not limit this period.

**Table 2.** Maximum Period Between Writing and Dispensing of Controlled Substance Prescriptions Allowed by Each State as of December 31, 2019

State	Maximum period, d^a^				
	Schedule II	Schedule III	Schedule IV	Schedule V	Overall limit for prescriptions in general
Alabama	No limit^b^	180	180	No limit^c^	No limit
Alaska	1 y^d^	180^e^	180^e^	1 y^f^	1 y
Arizona	90	180	180	1 y	1 y
Arkansas	180	180	180	180	1 y
California	180	180	180	180	No limit
Colorado	1 y^d^	180	180	180	1 y
Connecticut	No limit^b^	180	180	No limit^c^	No limit
Delaware	7	7	180	1 y^f^	1 y
District of Columbia	1 y^d^	180	180	1 y^f^	1 y
Florida	1 y^d^	180	180	180	1 y
Georgia	180	180	180	180	1 y
Hawaii	7	90	90	90	1 y
Idaho	No limit^b^	180	180	No limit^c^	No limit
Illinois	90	180	180	180	15 mo
Indiana	1 y^d^	180	180	180	1 y
Iowa	180	180	180	180	18 mo
Kansas	180	180	180	180	1 y
Kentucky	60	180	180	180	1 y
Louisiana	90	180	180	180	1 y
Maine	90	90	90	90	15 mo
Maryland	120	120	120	120	120
Massachusetts	30	30	30	No limit^c^	No limit
Michigan	90	180	180	1 y^f^	1 y
Minnesota	30	30^g^	30^g^	1 y^f^	1 y
Mississippi	180	180	180	180	1 y
Missouri	180	180	180	180	1 y
Montana	1 y^d^	180	180	180	1 y
Nebraska	180	180	180	180	1 y
Nevada	180	180	180	180	No limit
New Hampshire	180	180	180	180	1 y
New Jersey	30	30	30	30	1 y
New Mexico	180	180	180	180	1 y
New York	30	30	30	30	No limit
North Carolina	180	180	180	1 y^f^	1 y
North Dakota	180	180	180	180	1 y
Ohio	1 y^d^	180	180	1 y^f^	1 y
Oklahoma	30	180	180	180	1 y
Oregon	180	180	180	180	1 y
Pennsylvania	180	180	180	180	1 y
Rhode Island	90	180	180	180	1 y
South Carolina	90	180	180	180	2 y
South Dakota	No limit^b^	180	180	No limit^c^	No limit
Tennessee	1 y^d^	180	180	1 y^f^	1 y
Texas	21	180	180	180	1 y
Utah	30	180	180	1 y	1 y
Vermont^h^	30	180^e^	180^e^	1 y^f^	1 y
Virginia	180	180	180	180	1 y
Washington	180	180	180	180	1 y
West Virginia	90	180	180	180	1 y
Wisconsin	60	180	180	1 y^f^	1 y
Wyoming	180	180	180	2 y^f^	2 y

^a^ We considered laws that restricted the maximum period between writing and dispensing to 6 months vs 180 days to be the same (and similarly for laws restricting the maximum period to 3 months vs 90 days). Periods are presented in days unless indicated otherwise.

^b^ Alabama, Connecticut, Idaho, and South Dakota had no law regulating the maximum period between writing and dispensing of Schedule II controlled substances and also did not have a law regulating this period for prescriptions in general. Consequently, “no limit” is listed.

^c^ Alabama, Connecticut, Idaho, Massachusetts, and South Dakota had no law regulating the maximum period between writing and dispensing of Schedule V controlled substances and also did not have a law regulating this period for prescriptions in general. Consequently, “no limit” is listed for Schedule V controlled substances.

^d^ Alaska, Colorado, District of Columbia, Indiana, Montana, Ohio, and Tennessee had no law regulating the maximum period between writing and dispensing of Schedule II controlled substances but did have a law regulating this period for prescriptions in general. This latter period was 1 year in all 7 states, so “1 y” is listed. Florida had a law prohibiting dispensing of Schedule II to III controlled substances more than 14 days after a surgical procedure; outside of this situation, the maximum period between writing and dispensing of these substances is not specifically regulated. Because Florida prohibits dispensing of prescriptions in general beyond 1 year, “1 y” is listed.

^e^ Alaska and Vermont had no law for Schedule III and IV controlled substances, so we defaulted to the maximum time allowed between writing and dispensing for these substances under the Controlled Substances Act (180 days).

^f^ Alaska, Delaware, District of Columbia, Michigan, Minnesota, North Carolina, Ohio, Tennessee, Vermont, and Wisconsin had no law regulating the maximum period between writing and dispensing of Schedule V controlled substances but did have a law regulating this period for prescriptions in general. This latter period was 1 year in all of these states, so “1 y” is listed. Wyoming had no law regulating the maximum period between writing and dispensing of Schedule V controlled substances but did have a law regulating this period for prescriptions in general to 2 years, so “2 y” is listed.

^g^ Minnesota’s law (enacted on July 1, 2019, and repealed on March 29, 2020) only applied to Schedule II to IV narcotics such as opioids. The state also had a law restricting the maximum period between writing and dispensing of Schedule III to IV drugs to 180 days. We listed 30 days for Schedules II to IV.

^h^ Vermont had a law restricting the maximum period between writing and dispensing of prescriptions for extended-release oxycodone and hydrocodone to 7 days. Owing to the limited scope of this law, this is not reflected here.

### Impact of Minnesota’s Law

**Figure 2.**
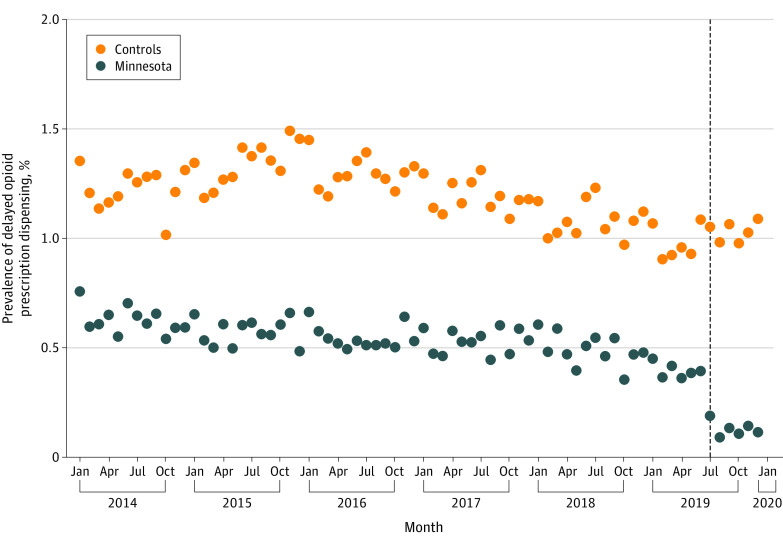
Changes in the Prevalence of Delayed Dispensing Among Opioid Prescriptions From Surgeons and Dentists After Enactment of Minnesota's Law The monthly prevalence of delayed dispensing among prescriptions for Schedule II to IV opioids from surgeons and dentists is compared in Minnesota vs 38 control states. The dashed vertical line denotes July 2019, the month during which Minnesota’s law was enacted to preclude dispensing of opioid prescriptions more than 30 days after writing.

During the pre- and postintervention periods, 0.55% and 0.13% of Schedule II to IV opioid prescriptions in Minnesota had delayed dispensing compared with 1.12% and 0.97% in control states. The enactment of Minnesota’s law in July 2019 was associated with a differential decrease of −0.22 percentage points (95% CI, −0.32 to −0.13 percentage points) in the prevalence of delayed dispensing compared with control states (Figure 2). Preintervention trends in the 2 comparison groups were similar. Results were unchanged in sensitivity analyses that used an alternative model specification, included a 1-month washout period, used alternative control states, or were limited to data from 2017 onward (eAppendix 6 in the Supplement).

## Discussion

In this cross-sectional analysis of a national pharmacy database representing 63% of US prescriptions, nearly 195 000 opioid prescriptions from US surgeons and dentists in 2019 were dispensed more than 30 days after writing. These results raise concerns that opioids prescribed by surgeons and dentists may sometimes be used for reasons or during a time frame other than that intended by the prescriber.

Delayed dispensing of opioid prescriptions from surgeons and dentists may be facilitated by lenient state laws regulating the maximum period between writing and dispensing of controlled substance prescriptions. As of December 2019, the maximum period between writing and dispensing of Schedule II drugs was either 180 days or 1 year in 26 states, whereas 4 states did not restrict this period at all. In the majority of states, the maximum period between writing and dispensing of prescriptions for Schedule III to IV drugs was 180 days.

In support of the notion that lenient state laws facilitate delayed dispensing, we observed that the prevalence of delayed dispensing among opioid prescriptions from surgeons and dentists decreased after a Minnesota law was enacted to preclude dispensing of prescriptions for Schedule II to IV opioids more than 30 days after writing. A potential concern is that this law could have decreased opioid access for patients with chronic pain. Perhaps owing to this concern, Minnesota’s law was repealed on March 29, 2020, at the beginning of the COVID-19 pandemic.^20^ To mitigate unintended consequences for patients with chronic pain, an option for policy makers is to enact laws limiting the maximum period between writing and dispensing of opioid prescriptions only when written by surgeons and dentists, who almost always prescribe opioids for acute pain. Such a law would differ from Florida’s law, which prohibits dispensing of prescriptions for Schedule II to III drugs more than 14 days after a surgical procedure, in that it would only affect opioid prescriptions and would index on the writing date instead of the procedure date. This approach could facilitate implementation by pharmacists, who can easily ascertain the writing date but typically lack access to patient medical records and information on procedure dates.

In addition to policy makers, other stakeholders could take steps to mitigate any prescription opioid misuse associated with delayed dispensing. For example, hospitals could alter their electronic health record systems so that the default signature for perioperative opioid prescriptions instructs pharmacists to refrain from dispensing the prescription more than 30 days after writing. Alternatively, clinicians could manually type or write these instructions in the signature. Insurers could also refuse to cover opioid prescriptions written by surgeons or dentists if they are tendered more than 30 days after writing.

Strengths of this study include the use of a national, all-payer pharmacy database. We also provide recent data describing the maximum period between writing and dispensing of controlled substance prescriptions allowed by each state. We used a strong quasi-experimental approach to evaluate whether Minnesota’s law shortening this period decreased delayed dispensing, which was less prone to confounding compared with a cross-sectional analysis examining the association between these periods and the prevalence of delayed dispensing in each state. Finally, although not related to the study’s primary purpose, our finding that 92.1% of opioid prescriptions from surgeons and dentists were dispensed within 3 days of writing may assist in informing decisions on the definition of perioperative opioid prescriptions in surgical and dental health services research.^21,22,23,24^

### Limitations

This study has some limitations. First, the lack of clinical details in the national pharmacy database precluded determination of whether delayed dispensing represented misuse. For example, some delayed dispensing events might reflect prescriptions intended for postprocedural pain but written at preoperative visits that occurred well before surgery. We reiterate, however, that our goal was to estimate the number of opioid prescriptions from surgeons and dentists with a potentially concerning dispensing pattern, not the number of opioid prescriptions definitively associated with misuse.

Second, analyses excluded opioid prescriptions from physician assistants and nurse practitioners, who account for one-fifth of perioperative opioid prescriptions.^25^ This exclusion was necessary because our database does not report whether prescriptions written by these clinicians were for surgical or dental care. As a result, these analyses likely underestimate the number of perioperative opioid prescriptions with delayed dispensing. Third, we deliberately used a stringent definition of delayed dispensing. In our database, 486 618 opioid prescriptions (2.3%) from surgeons and dentists in 2019 were dispensed more than 14 days after writing, a pattern that could also be concerning. Finally, owing to data limitations, we could not determine whether delayed dispensing was more prevalent among patients with chronic pain or prior opioid use; the latter could not be accurately measured because the study database does not capture dispensing from all US pharmacies.

## Conclusions

Delayed dispensing of surgical and dental opioid prescriptions, although uncommon in a relative sense, occurs hundreds of thousands of times per year owing to the volume of opioid prescribing by US surgeons and dentists. Future studies should examine whether there is a link between delayed dispensing and prescription opioid misuse using alternative data sources and approaches, including qualitative methods.

## Notes

Supplement. **eAppendix 1.** List of Opioid Analgesics Included **eAppendix 2.** Search Strategy for State Laws **eAppendix 3.** List of Laws Regulating the Maximum Period Between Writing and Dispensing of Controlled Substance Prescriptions **eAppendix 4.** Prevalence of Delayed Dispensing Among Opioid Prescriptions From Surgeons and Dentists in 2019 by State, Using IQVIA Formulary Impact Analyzer Data **eAppendix 5.** Prevalence of Delayed Dispensing Among Opioid Prescriptions Dispensed in 2019, by Specialty **eAppendix 6.** Sensitivity Analyses for Difference-in-Differences Analyses
