# Supplementary material for: Estimation of the Prevalence of Delayed Dispensing Among Opioid Prescriptions From US Surgeons and Dentists
Source: JAMA Netw Open. 2022 May 27;5(5):e2214311. doi: 10.1001/jamanetworkopen.2022.14311 (PMC9142869; doi:10.1001/jamanetworkopen.2022.14311)
Supplement: Supplement. — eAppendix 1. List of Opioid Analgesics Included eAppendix 2. Search Strategy for State Laws eAppendix 3. List of Laws Regulating the Maximum Period Between Writing and Dispensing of Controlled Substance Prescriptions eAppendix 4. Prevalence of Delayed Dispensing Among Opioid Prescriptions From Surgeons and Dentists in 2019 by State, Using IQVIA Formulary Impact Analyzer Data eAppendix 5. Prevalence of Delayed Dispensing Among Opioid Prescriptions Dispensed in 2019, by Specialty eAppendix 6. Sensitivity Analyses for Difference-in-Differences Analyses [file jamanetwopen-e2214311-s001.pdf]

Supplementary Online Content

Chua KP, Waljee JF, Smith MA, Bahl S, Nalliah RP, Brummett CM. Estimation of the prevalence of delayed dispensing among opioid prescriptions from US surgeons and dentists. *JAMA Netw Open*. 2022;5(5):e2214311. doi:10.1001/jamanetworkopen.2022.14311

eAppendix 1. List of Opioid Analgesics Included
eAppendix 2. Search Strategy for State Laws
eAppendix 3. List of Laws Regulating the Maximum Period Between Writing and Dispensing of Controlled Substance Prescriptions
eAppendix 4. Prevalence of Delayed Dispensing Among Opioid Prescriptions From Surgeons and Dentists in 2019 by State, Using IQVIA Formulary Impact Analyzer Data
eAppendix 5. Prevalence of Delayed Dispensing Among Opioid Prescriptions Dispensed in 2019, by Specialty
eAppendix 6. Sensitivity Analyses for Difference-in-Differences Analyses

This supplementary material has been provided by the authors to give readers additional information about their work.

© 2022 Chua KP et al. *JAMA Network Open*.

eAppendix 1. List of Opioid Analgesics Included

Opioid analgesics included benzhydrocodone, buprenorphine, butorphanol, codeine, dihydrocodeine, fentanyl, hydrocodone, hydromorphone, levorphanol, meperidine, methadone, morphine, nalbuphine, opium, oxycodone, oxymorphone, pentazocine, propoxyphene, sufentail, tapentadol, and tramadol. Buprenorphine formulations approved for opioid use disorder were excluded, as were opioid cough-and-cold medications.

© 2022 Chua KP et al. *JAMA Network Open*.

eAppendix 2. Search Strategy for State Laws

<u>Laws regulating maximum period between writing and dispensing of controlled substance prescriptions</u>

During winter 2021, we used NABPLAW Online to compile all state laws regulating the maximum period between writing and dispensing of controlled substance prescriptions that were effective at the time of the search. We started with NABPLAW Online as opposed to WestLaw Edge because the former only contained pharmacy laws, making the relevant laws easier to identify. We used the following search terms in NABPLAW for each of the 50 states and the District of Columbia (the example below is for Alabama):

- "controlled substance" "day" "AL"
- "controlled substance" "month" "AL"
- "controlled substance" "year" "AL"
- "Schedule II" "day" "AL"; "Schedule II" "month" "AL"; "Schedule II" "year" "AL"
- "Schedule III" "day" "AL"; "Schedule III" "month" "AL"; "Schedule III" "year" "AL"
- "Schedule IV" "day" "AL"; "Schedule IV" "month" "AL"; "Schedule IV" "year" "AL"
- "Schedule V" "day" "AL"; "Schedule V" "month" "AL"; "Schedule V" "year" "AL"

Of 180 state laws identified, we excluded laws that only applied to partial refills, laws that only applied to patients in long-term care facilities, laws that only apply to disciplinary measures for violations of regulations surrounding controlled substance prescribing regulations, and laws that were not applicable to the pharmacy setting. The 96 remaining laws all pertained to the maximum period between writing and dispensing of controlled substance prescriptions.

Although NABPLAW included the text of the current 96 laws along with the dates of amendments, it did not contain the text of the amendments. Therefore, we used Thomas Reuters Westlaw Edge to compile the text and effective dates of all amendments. By comparing the text of the law effective as of the time of the search and any amendments, we determined which laws were effective as of December 31, 2019 and whether states had passed laws regulating the maximum period between writing and dispensing of controlled substance prescriptions between 2014-2019 (for the difference-in-differences analysis).

We conducted two additional checks to ensure that we had identified all relevant laws. First, we used the same search terms as above in WestLaw Edge and obtained the same set of laws. Second, we used more general search terms to search for relevant laws for each state in WestLaw Edge: "controlled substance", "Schedule II", "Schedule III", "Schedule IV", "Schedule V." This strategy yielded the same set of laws as in our original search of NABPLAW.

<u>Laws regulating maximum period between writing and dispensing of non-controlled substance prescriptions</u>

When states did not have specific laws regulating the maximum period between writing and dispensing of controlled substances in DEA Schedule III-IV, we set the maximum period to 6 months, the maximum allowed under the Controlled Substance Act.

When states did not have specific laws regulating the maximum period between writing and dispensing of controlled substances in DEA Schedule II or V, we set the maximum period to the period allowed for non-controlled substance prescriptions by the state. To identify state laws regulating this period for non-controlled substance prescriptions, we used WestLaw Edge and additionally contacted state pharmacy associations.

© 2022 Chua KP et al. *JAMA Network Open*.

eAppendix 3. List of Laws Regulating the Maximum Period Between Writing and Dispensing of Controlled Substance Prescriptions

State	Law
Alabama	Ala.Code 1975 § 20-2-58
Alaska	AK Rule 12 AAC 52.470
Arizona	A.R.S. § 36-2525<br>A.R.S. § 32-1968
Arkansas	Ark. Admin. Code 070.00.7-07-04-0004<br>Ark. Admin. Code 007.07.2-II-IX<br>Ark. Admin. Code 007.07.2-II-VIII<br>A.C.A. § 5-64-308<br>AR Rule 070.00.7-07-02-0002
California	West's Ann.Cal.Health & Safety Code § 11200<br>West's Ann.Cal.Health & Safety Code § 11166
Colorado	C.R.S.A. § 18-18-308<br>CO Rule 719-1:3.00.00
Connecticut	C.G.S.A. § 21a-249
Delaware	24 Del. Admin. Code CSA 4.0<br>16 Del.C. § 4739<br>DE Rule 2500-5.0
D.C.	DC ST § 48-903.08<br>22-B DCMR § **1310**<br>Section 22-B1325., 1325.8
Florida	West's F.S.A. § 465.0276<br>West's F.S.A. § 893.04<br>Rule 64B16-27.211, F.A.C.<br>FL Rule 64B16-27.211
Georgia	Ga Comp. R. & Regs. 480-21-.05<br>Ga Comp. R. & Regs. 480-22-.08<br>Ga. Code Ann., § 16-13-41<br>Ga. Code Ann., § 26-4-80<br>GA Rule 480-22-.12
Hawaii	HRS § 329-38<br>HI Law 328-16<br>Haw. Admin. Rules (HAR) § 23-200-15
Idaho	I.C. § 37-2722
Illinois	720 ILCS 570/312<br>410 ILCS 620/2.36
Indiana	856 IAC 2-6-13<br>IC 35-48-3-9<br>IN Rule 856 IAC Rule 1-32-2
Iowa	I.C.A. § 124.308<br>Iowa Admin. Code 657-10.24(124,126,155A)<br>Iowa Admin. Code 657-10.32(124)<br>IA Rule 657-7.11. (124,126,155A)<br>IA Law 155A.29
Kansas	K.A.R. 68-20-19<br>KAR 68-20-20<br>K.S.A. 65-4123

© 2022 Chua KP et al. *JAMA Network Open*.

	K.S.A. 65-1637
Kentucky	KY Law 218A.180<br>201 KAR 2:185
Louisiana	La. Admin Code. tit. 46, Pt LIII, § 2745<br>LSA-R.S. 40:978<br>La. Admin Code. tit. 46, Pt LIII, § 2747<br>La. Admin Code. tit. 46, Pt LIII, § 2525<br>**La**. Admin Code. tit. 50, Pt XXIX, § 117
Maine	02-392 CMR Ch. 19, § 2<br>02-392 CMR Ch. 19, § 5
Maryland	COMAR 10.19.03.08<br>COMAR 10.19.03.09<br>MD Code, Health Occupations, § 12-503.
Massachusetts	M.G.L.A. 94C § 23<br>M.G.L.A. 94C § 18
Michigan	M.C.L.A. 333.7333<br>Mich. Admin. Code R 338.584
Minnesota	M.S.A. § 152.11<br>Law 151.211<br>Minnesota Rules, part 6800.3510
Mississippi	30 Miss. Admin. Code Pt. 3001, Art. XIX<br>30 Miss. Admin. Code Pt. 3001, Art. XII<br>Miss. Code Ann. § 41-29-137<br>30 Miss. Admin. Code Pt. 3001, Art. XXI<br>30 Miss. Admin. Code Pt. 2640, R. 1.11
Missouri	V.A.M.S. 195.060<br>MO Rule 19 CSR 30-1.064<br>20 CSR 2220-2.110
Montana	MCA 50-32-208<br>Mont.Admin.R. 24.174.831
Nebraska	Neb.Rev.St. § 28-414<br>Neb. Admin. R. & Regs. Tit. 175, Ch. 8, § 006<br>Neb.Rev.St. § 28-414.01<br>Neb.Rev.St. § 38-2870<br>NE Rule 175-8-006
Nevada	N.R.S. 453.431<br>N.R.S. 639.2393<br>N.R.S. 453.256
New Hampshire	N.H. Rev. Stat. § 318-B:9<br>N.H. Code Admin. R. Ph 704.14
New Jersey	N.J.A.C. 13:45H–7.5<br>N.J.A.C. 13:45H–7.14<br>N.J.S.A. 24:21–15<br>N.J.A.C. 13:39–7.3<br>NJ Rule 13-45H-7.15
New Mexico	N.M. Admin. Code 16.19.20.45<br>N. M. S. A. 1978, § 30-31-18<br>N. M. S. A. 1978, § 26-1-16
New York	10 NYCRR 80.73<br>McKinney's Public Health Law § 3333

© 2022 Chua KP et al. *JAMA Network Open*.

	10 NYCRR 80.78<br>10 NYCRR 80.69<br>10 NYCRR 80.74<br>NY Law 3339
North Carolina	N.C.G.S.A. § 90-106<br>N.C.G.S.A. § 90-85.32
North Dakota	NDCC, 19-03.1-22<br>NDCC, 19-02.1-15
Ohio	R.C. § 3719.05<br>R.C. § 4729.281
Oklahoma	Okla. Admin. Code 475:30-1-4<br>Okla. Admin. Code 475:30-1-11<br>63 Okl.St.Ann. § 2-309<br>OK Rule 535:15-3-12<br>OK Rule 535:15-3-11
Oregon	OAR 855-041-1125
Pennsylvania	49 Pa. Code § 27.18<br>35 P.S. § 780-111
Rhode Island	Gen.Laws 1956, § 21-28-3.18<br>216-RICR- 40-15-**1**.4
South Carolina	Code 1976 § 44-53-360<br>S.C. Code of Regulations R. 61-4.1102<br>S.C. Code of Regulations R. 61-4.1202<br>Code 1976 § 40-43-86
South Dakota	ARSD 44:58:08:17
Tennessee	T. C. A. § 53–11–308<br>Tenn. Comp. R. & Regs. 1140-03-.03
Texas	22 TAC § 315.3<br>22 TAC § 315.5<br>V.T.C.A., Health & Safety Code § 481.074<br>22 TAC § 291.34
Utah	U.C.A. 1953 § 58-37-6<br>U.A.C. R156-37-603<br>U.C.A. 1953 § 58-17b-609
Vermont	Vt. Admin. Code 20-4-1400:10.16<br>Vt. Admin. Code 12-5-53:8.0<br>Vt. Admin. Code 20-4-1400:10.17
Virginia	18 VAC 110-20-290<br>18 VAC 110-20-320
Washington	West's RCWA 69.50.308<br>WAC 246-887-020<br>WAC 246-945-012
West Virginia	W. Va. Code St. R. § 15-2-8<br>W. Va. Code St. R. § 15-1-7<br>WV Law 60A-3-308
Wisconsin	Wis. Adm. Code § Phar 8.05<br>W.S.A. 961.38<br>Wis. Adm. Code § Phar 8.06<br>Wis. Adm. Code § Phar 7.03
Wyoming	WY Rules and Regulations 059.0002.6 § 13

© 2022 Chua KP et al. *JAMA Network Open*.

	WY Rules and Regulations 059.0002.6 § 20<br>WY Rule 059.0002.10 Sec. 7<br>WY Rule 059.0002.10 Sec. 3<br>W.S.1977 § 33-24-101

© 2022 Chua KP et al. *JAMA Network Open*.

eAppendix 4. Prevalence of Delayed Dispensing Among Opioid Prescriptions From Surgeons and Dentists in 2019 by State, Using IQVIA Formulary Impact Analyzer Data

**Group**	**Number of prescriptions dispensed in 2019**	**Number of prescriptions with delayed dispensing (% of all prescriptions in state)**
**All prescriptions in sample**	20,858,413	194,452 (0.9%)
**State**		
Alabama	737,176	13,989 (1.9%)
Alaska	54,338	209 (0.4%)
Arizona	450,399	2,913 (0.6%)
Arkansas	419,950	7,082 (1.7%)
California	1,339,879	18,778 (1.4%)
Colorado	526,996	7,693 (1.5%)
Connecticut	125,490	343 (0.3%)
Delaware	58,675	97 (0.2%)
District of Columbia	23,948	189 (0.8%)
Florida	1,389,461	19,110 (1.4%)
Georgia	1,006,683	13,337 (1.3%)
Hawaii	30,926	40 (0.1%)
Idaho	173,963	935 (0.5%)
Illinois	813,048	8,704 (1.1%)
Indiana	439,111	2,769 (0.6%)
Iowa	196,543	936 (0.5%)
Kansas	255,647	2,574 (1.0%)
Kentucky	504,036	2,316 (0.5%)
Louisiana	657,254	9,911 (1.5%)
Maine	48,215	52 (0.1%)
Maryland	258,092	2,471 (1.0%)
Massachusetts	167,121	376 (0.2%)
Michigan	603,135	4,581 (0.8%)
Minnesota	245,053	643 (0.3%)
Mississippi	355,501	2,598 (0.7%)
Missouri	623,077	3,830 (0.6%)
Montana	83,873	463 (0.6%)
Nebraska	134,448	671 (0.5%)
Nevada	161,227	698 (0.4%)
New Hampshire	56,407	514 (0.9%)
New Jersey	350,786	533 (0.2%)
New Mexico	140,668	1,789 (1.3%)
New York	577,659	331 (0.1%)
North Carolina	611,043	6,463 (1.1%)
North Dakota	43,026	215 (0.5%)
Ohio	812,293	2,221 (0.3%)
Oklahoma	514,909	497 (0.1%)
Oregon	343,910	5,857 (1.7%)
Pennsylvania	571,177	6,409 (1.1%)
Rhode Island	28,040	127 (0.5%)

© 2022 Chua KP et al. *JAMA Network Open*.

South Carolina	367,540	3,574 (1.0%)
South Dakota	65,017	218 (0.3%)
Tennessee	772,784	6,355 (0.8%)
Texas	1,884,032	13,179 (0.7%)
Utah	274,141	1,613 (0.6%)
Vermont	23,127	229 (1.0%)
Virginia	423,219	5,750 (1.4%)
Washington	593,296	7,244 (1.2%)
West Virginia	131,476	1,317 (1.0%)
Wisconsin	333,971	1,372 (0.4%)

© 2022 Chua KP et al. *JAMA Network Open*.

eAppendix 5. Prevalence of Delayed Dispensing Among Opioid Prescriptions Dispensed in 2019, by Specialty

Group	Number of prescriptions dispensed in 2019[a]	Number of prescriptions with delayed dispensing (% of all prescriptions in specialty)
**All prescriptions in sample**	101,991,162	5,915,854 (5.8%)
**Specialty**		
Emergency medicine	3,649,507	25,063 (0.7%)
Family medicine	19,427,204	1,392,974 (7.1%)
Internal medicine	12,309,427	693,048 (5.6%)
Nurse practitioner	13,068,710	821,951 (6.3%)
Osteopathy	13,601	336 (2.5%)
Pain medicine	7,699,959	975,473 (12.7%)
Physician assistant	9,793,181	536,523 (5.5%)
Other	15,120,336	1,275,508 (8.4%)
Unknown	50,824	526 (1.0%)
Surgery	13,214,367	127,786 (1.0%)
Dentistry	7,644,046	66,666 (0.9%)

[a]These prescriptions met all study inclusion and exclusion criteria (e.g., they were not refills of prior prescriptions) except that they were written by any prescriber, as opposed to surgeons and dentists specifically.

© 2022 Chua KP et al. *JAMA Network Open*.

eAppendix 6. Sensitivity Analyses for Difference-in-Differences Analyses

For prescription *i* dispensed in state *s* during month t, the model specification in the main analysis was:

$E(Y_{ist}) = \beta_{1*}State_s + \beta_{2*}Month_t + \beta_3 \, Minnesota_{ist*}Post_{ist}$

Where Y_{ist} is an indicator that equals 1 if the prescription was dispensed more than 30 days after writing and 0 otherwise, Minnesota is an indicator that equals 1 if the prescription was dispensed to a patient residing in Minnesota and 0 if dispensed to a patient in a control state, and Post is an indicator that equals 1 if the prescription was dispensed on or after July 1, 2019 (the date on which Minnesota's law was enacted) and 0 otherwise. State is a vector of state fixed effects (accounting for time-invariant confounders at the state level) and month is a vector of year-month fixed effects (accounting for confounders associated with specific time periods). Models employed standard errors clustered at the state level.

Analyses excluded nine states that restricted the maximum period between writing and dispensing of prescriptions for Schedule II, III, or IV drugs to 30 days or less. Analyses also excluded Louisiana, New Mexico, and Florida, the last of which prohibited dispensing of prescriptions for Schedule II-III drugs more than 14 days after the date of a surgical procedure. The remaining 38 states were control states.

We conducted several sensitivity analyses, all of which yielded similar difference-in-differences estimates as in the main analysis (-0.22 percentage points, 95% CI: -0.32, -0.13).

- Controlled for patient age, patient sex, DEA Schedule, whether prescription was written by a surgeon or dentist, and prescription method of payment: -0.23 (-0.33, -0.14).
- Used standard errors clustered at the patient level instead of state level: -0.22 (-0.25, -0.20).
- Limited to data from 2017-2019: -0.22 (-0.32, -0.13)
- Excluded data from July 2019 as a wash-out period: -0.23 (-0.33, -0.14)

We also constructed an alternative control group comprised of the 9 states that restricted the maximum period between writing and dispensing of prescriptions for Schedule II, III, or IV drugs to 30 days or less throughout the study period. Using the same model specification as in the primary analysis, the difference-in-differences estimate was -0.34 (-0.38, -0.29).

© 2022 Chua KP et al. *JAMA Network Open*.
